# The Prediction of Compressive Strength and Compressive Stress–Strain of Basalt Fiber Reinforced High-Performance Concrete Using Classical Programming and Logistic Map Algorithm

**DOI:** 10.3390/ma15196975

**Published:** 2022-10-08

**Authors:** Mohammad Hematibahar, Nikolai Ivanovich Vatin, Hayder Abbas Ashour Alaraza, Aghil Khalilavi, Makhmud Kharun

**Affiliations:** 1 Department of Civil Engineering, Academy of Engineering, Peoples’ Friendship University of Russia (RUDN), 6 MiklukhoMaklaya Street, 117198 Moscow, Russia; 2 Peter the Great St. Petersburg Polytechnic University, 195251 St. Petersburg, Russia; 3 Department of Civil Engineering, Engineering College, University of Kerbala, J253+VG7, Karbala 56001, Iraq; 4 Department of Mathematics, Tarbiat Modares University, Jalal Al Ahmad Street, P9FM+9H, Tehran 14115-111, Iran; 5 Department of Reinforced Concrete and Stone Structures, Moscow State University of Civil Engineering, 26 Yaroslavskoye Highway, 129337 Moscow, Russia

**Keywords:** basalt fiber high-performance concrete, compressive strength prediction, compressive stress–strain curve, logistic function and map, classical programming

## Abstract

In this research, the authors have developed an algorithm for predicting the compressive strength and compressive stress–strain curve of Basalt Fiber High-Performance Concrete (BFHPC), which is enhanced by a classical programming algorithm and Logistic Map. For this purpose, different percentages of basalt fiber from 0.6 to 1.8 are mixed with High-Performance Concrete with high-volume contact of cement, fine and coarse aggregate. Compressive strengths and compressive stress–strain curves are applied after 7-, 14-, and 28-day curing periods. To find the compressive strength and predict the compressive stress–strain curve, the Logistic Map algorithm was prepared through classical programming. The results of this study prove that the logistic map is able to predict the compressive strength and compressive stress–strain of BFHPC with high accuracy. In addition, various types of methods, such as Coefficient of Determination (R2), are applied to ensure the accuracy of the algorithm. For this purpose, the value of R2 was equal to 0.96, which showed that the algorithm is reliable for predicting compressive strength. Finally, it was concluded that The Logistic Map algorithm developed through classical programming could be used as an easy and reliable method to predict the compressive strength and compressive stress–strain of BFHPC.

## 1. Introduction

High-Performance Concrete (HPC) is a type of concrete with minimal penetration of aggressive agents. It has become very popular in recent years because it surpasses conventional concrete in terms of its physical and chemical properties. HPC can be enhanced in terms of durability and mechanical properties. Therefore, it is mostly used in important structures such as tall buildings, bridges, runways, and highway pavements. High-performance concrete (HPC) is a type of concrete with minimal penetration of aggressive agents, and it has become very popular in recent years because it surpasses conventional concrete in terms of its mechanical and chemical properties [1,2,3]. The effect of basalt fiber (BF) on HPC was investigated in many previous types of research [4,5,6,7,8,9,10,11]. In fact, BF was added to HPC to increase the durability, tensile, and flexural strengths [12,13,14]. For example, Kharun et al. [5] added basalt fiber to High-Strength Concrete (HSC) to enhance tensile and flexural strength. Their results illustrated that the BF-reinforced HSC (BFHSC) was more durable than conventional HSC. Furthermore, their results show that the addition of 1.2% BF to HSC can improve the compressive strength by more than 37% and the tensile strength by more than 70%. In fact, the results of adding BF to HSC and HPC are shown by the significant increase in flexural and tensile strength and the improvement of concrete durability [5,7,15]. Moreover, including mechanical aspects, BF has economic aspects as an alternative material to increase the mechanical properties of HPC [16]. For example, Hematibahar [17] investigated adding BF to HPC. The researchers examined the effect of adding different fractions of BF content between 0.6 and 1.8 to the HPC. Finally, he found that adding 1.2% of BF had a maximum effect on the compressive and flexural strengths. According to his results, the compressive strength improved to 102.3 MPa from 101.43 MPa when 1.2% BF was added. In addition, by adding 1.2% BF to HPC, the flexural strength increased by more than 35 percent. According to the results of Ayub et al. [7], 2% of BF had a maximum effect on the mechanical behavior of HPC, such as compressive, tensile, and flexural strength. Ayub et al. [7] showed that the compressive strength of BFHPC with 2% BF increased by more than 2%, and by adding 3% BF to High-Performance Concrete, the tensile strength improved by more than 17%. Another study by Mohaghegh et al. [18] analyzed the mixing of different percentages of BF into HPC for testing the shear capacity of concrete beams. They believed that the mechanical properties of HPC are related to the BF dosage. They represented that 1.33% of BF had the best effect on the mechanical properties. Additionally, they proved that a high dosage of BF was effective in the shear capacity of HPC. Overall, BF was an effective variable for improving the mechanical properties of HPC. Another method that engineers use to improve the mechanical properties of HPC is to add chemical powders. For example, the silica fume can fill the voids between the BF and cement matrix [19]. Their results show that the silica fume was effective in enhancing the compressive, flexural, and tensile strength of HPC.

Currently, researchers tend to practice computer programming such as machine learning, deep learning, and other types to predict the mechanical characteristics of concrete. Mechanical computer prediction can reduce the budget of experiments and waste materials and decrease time wastage. However, there are many examples of using Artificial Neural Networks (ANN), Fuzzy Logic (FL), Linear Regression (LR), Multiple Linear Regression (MLR), etc., but a simple and comprehensive programming code to predict mechanical properties is a problem. For example, Shahmansouri et al. [20] developed an Algorithm programming method for predicting natural zeolitic concrete compressive strength in different curing period ages. They used more than 56 mixture designs as statistic samples to find the best result. They used Root Mean Square Errors (MSE) to find the accuracy of the developing program. Their results show that the MSE value for compressive strength was 1.6, while the current study had a better MSE result. They concluded that the prediction of compressive strength helps to save time in the testing process. ANN is used as a mechanical properties forecast method in various studies considering mixing design properties [21,22,23]. For example, Chopra et al. [24] estimated the mechanical properties of concrete using ANNs. They collected compressive strength results for 21-, 56-, and 91-days curing periods. They applied the ANNs using the Leveberg–Marquardt (LM) training function. Their results show that the prediction of compressive strength was reliable so the Coefficient of Determination (R2) was more than 0.8. However, the complicated programming method and the use of the LM method cannot be justified for general engineers, and it is another gap in simplifying the calculation method. In another example, Topcu and Saridemir [25] studied a concrete mixture with fly ash using the ANNs and FL methods separately. They focused on aggregate size to predict compressive strength. They concluded that the prediction of the mechanical properties of concrete was successful, so that R2 calculated for all samples was greater than 0.9. According to their prediction results, the FL and ANNs methods are acceptable for comparing the experimental results. Reza Kashyzadeh et al. [26] also used the Data Mining method to determine the compressive strength of concrete, considering the shape and size of the dry aggregate. They developed an algorithm to find the compressive strength of ordinary concrete through the Data Mining of the dry temperature and aggregate shapes. They found that the input parameters, such as aggregate size and shape, affect compressive strength. In addition, they found that the cold wind process in the drying of aggregates has the greatest effect on increasing the compressive strength so that the compressive strength in the cold wind process is improved by more than 8%. Moreover, Kashizadeh et al. [18] investigation was related to the aggregate type. They used more than 108 cubic specimens to predict the compressive strength. They found that the compressive strength increased by more than 30% when the cubic samples were cured at 15–5 °C. In fact, most research is aimed at finding compressive strength as mechanical properties through ML and ANN algorithms.

Not only is the existence of a simple prediction of the concrete mechanical properties a fundamental issue, but also the lack of simulating the compressive stress–strain curve through simple equations and formulations is another gap. Nevertheless, some researchers are trying to simulate the compressive stress–strain curves [27]. For example, Carreira and Chu [28] proposed a model for simulating the compressive stress–strain curve. Although their results performed well, predicting the plastic phase of concrete had a problem. Moreover, Ezeldin and Balaguru [29] provided another method to simulate the compressive stress–strain curve. Their results were more accurate than other previous methods. The lack of simple prediction formulas and the difficulty of these algorithms became the reason for studying this research and presenting a new simple method.

This research investigates a new approach for predicting the mechanical properties of concrete. The main idea was the link between Logistic Function to predict the compressive strength, Logistic Map for simulating the compressive stress–strain curves, and classical programming. The method can predict the compressive strength and compressive stress–strain curves through the Classical Programming Logistic Map Algorithm (CPL). BFHPC was chosen for this study. This Algorithm can be checked with real test results and a comparison of the experimental data with the simulation and estimated results. In general, the equations and Algorithms are capable of predicting the compressive strength and the compressive stress–strain curve with high accuracy and using a simple classical programming method.

## 2. Materials and Methods

**Figure 1:**
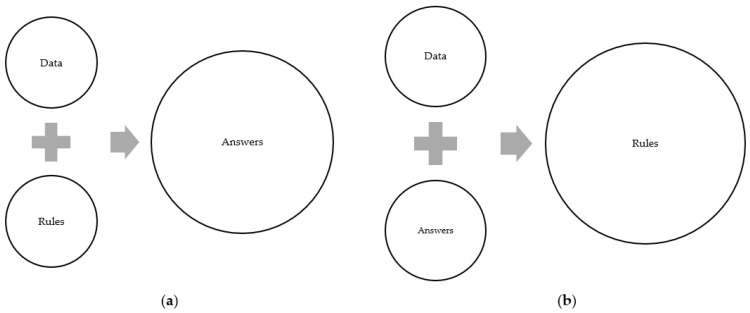
The Algorithm of different programming types: (**a**) the CP method; (**b**) the AI programming method.

Based on the purpose of the study, Classical Programming was employed to predict the compressive strength and the compressive stress–strain curve. Classical Programming (CP) was applied to the data and rules as inputs to find answers (Figure 1a).

Moreover, the data and answers are defined as the inputs in Artificial Intelligence (AI) (Figure 1b). The inputs and results are the main differences between Classical Programming and AI.

Generally, the Classical Programming method is a simple way to predict the mechanical properties of concrete as the answers. At the same time, AI needs a more complicated Algorithm for prediction. In this study, Classical Programming is chosen to predict the compressive strength and the compressive stress–strain curves.

### 2.1. Materials

#### Experimental Materials and Mixing Method

**Figure 2:**
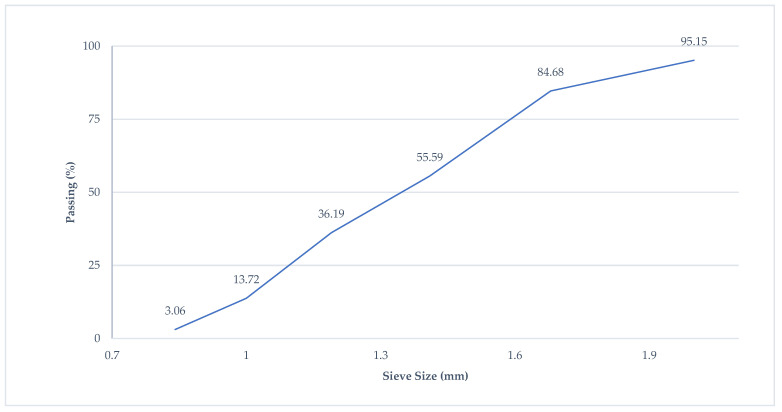
Granulometric Curves of Coarse Aggregates.

**Figure 3:**
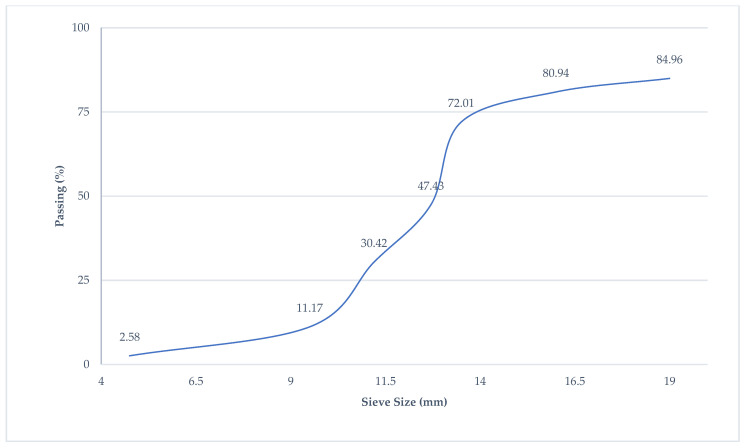
Granulometric Curves of Fine Aggregates.

**Figure 4:**
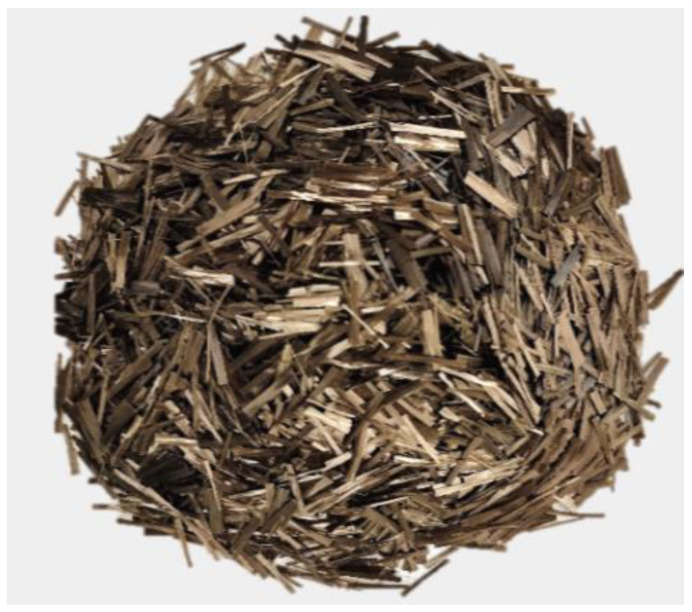
Chopped Basalt Fibers.

In order to prepare the BFHPC samples, the following material was used: Portland cement (OPC), quartz sand, crushed granite, quartz flour, micro silica, superplasticizer, tap water, and BF [4,17]. The specific gravity of Cement M500 was 1756 Kg/m^3^. The initial setting time of the cement was 60 min, and the final setting time was 600 min. Table 1 shows the chemical compounds of Cement M500 manufactured by Novoroscement factory, Novorossiysk, Russia. The crushed granite with a particle size range of 20 mm to 5 mm with specific gravity (2853 Kg/m^3^), and the granulometric curves of the coarse aggregates is shown in Figure 2. Quartz sands with a particle size range of 0.8 mm to 2.0 mm were mixed with concrete, and they were collected from the Ryazan region. The specific gravity of the fine aggregates was 2384 Kg/m^3^, and the granulometric curves are illustrated in Figure 3. Two kinds of aggregates were produced by the SUKHOGRUZ Company. SILVERBOND 50 quartz flour manufactured by SIBELCO in the Antwerp, Belgiumwas used. Micro-silica-type MK85 from the Novolipetsk steel company (NLMK) in Lipetsk, Russia was added to the concrete type to cover the holes between the aggregate and cement paste (Table 2, the properties of micro silica). The addition of silica to concrete can provide concrete properties with a high density. Moreover, the smaller silica grain size causes the higher density of the concrete, which is beneficial for the preparation of BFHPC [17]. Kamenny Vek’s chopped BF was used as the additional fibers (Figure 4 and Table 3).

The composition of the BFHPC design mixture is shown in Table 4. In the previous study, the percentage of BF 0.6, 0.9, 1.2, 1.5, and 1.8 percentages were investigated. BFs were the only additional variable material. The purpose of this study was to determine the effective percentage of additional BF to improve HPC while other materials were kept constant.

**Figure 5:**
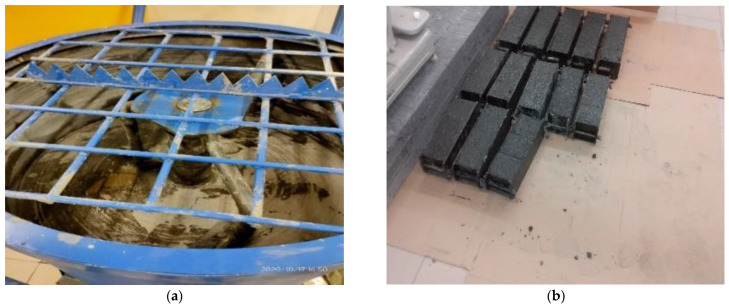

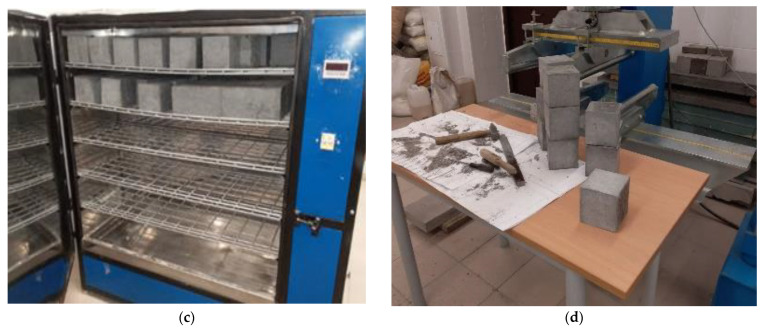
Mixing design devices: (**a**) Pan Mixer; (**b**) Concrete formwork; (**c**) Moist Cabinet; (**d**) cleaning method.

To prepare BHPC, concrete pan mixers with a constant speed of 48 rpm have been used for mixing materials (Figure 5a). In this case, first, the two types of aggregates were mixed by a 133-L mixer for about 2 min, then water was added to the cement. Additionally, the concrete was mixed for about 2 min after adding the chemical powders. Thus, the concrete was installed in the formwork. The compression testing formwork was $(100\times 100\times {100)\;(\mathrm{mm}}^{3})$ cubic concrete, and flexural three-point flexural formwork was $(100\times 100\times {600)\;(\mathrm{mm}}^{3})$ prisms (Figure 5b). The formwork was then molded underwater at a temperature of more than 50 °F for 7, 14, and 28 days during the curing period (Figure 5c). In the end, the cubes are cleaned before the compression test. Cleaning the cubes helped to achieve the best and most accurate compressive strength results (Figure 5d).

The compression test and flexural strength samples were tested according to GOST (GOST 10180-2012 Concretes. Methods for strength), which was applied for the test of the cube compressive strength in Russia and ASTM C293/C293, ASTM C1202 (American Society for Testing and Materials) standards. The compressive strength tests were performed for 7, 14, and 28 days with three different samples for each day [30,31,32].

**Figure 6:**
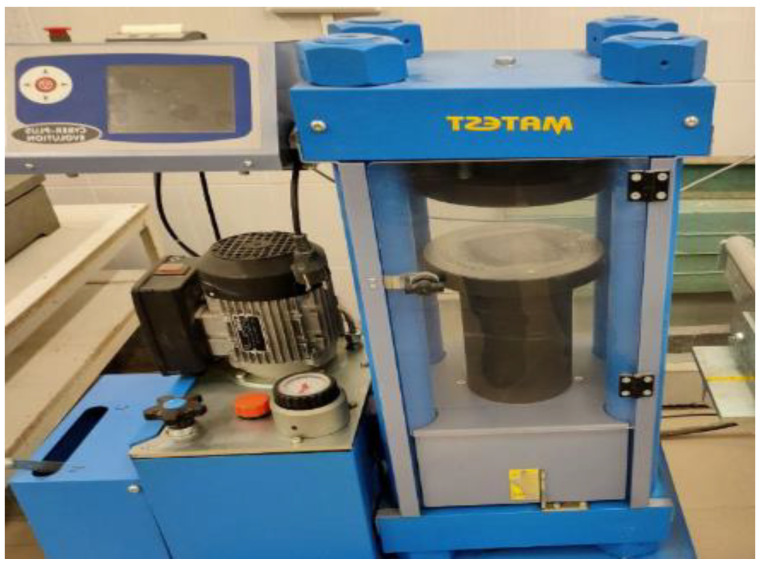
The compression test machine (C025N Matest Machine).

The Matest C025N manufactured by Mates cimoany, Bergamo, Italy device was used to set the compressive and flexural strength tests. The Matest C025N machine has a compression capacity of 1300 KN. In addition, the Matest C025N model uses a motorized system to apply the load, and the pressure rate was 400,000 MPa/s. Moreover, the computational compressive strain method was based on the energy absorption measurements (Figure 6).

### 2.2. Mathematical Modeling

Due to the prediction compressive strength of BFHPC, new Classical Programming is represented in this study. For this purpose, the Logistic Function predicted compressive strength, and the Logistic Map was defined by simulating the compressive stress–strain curves.

**Figure 7:**
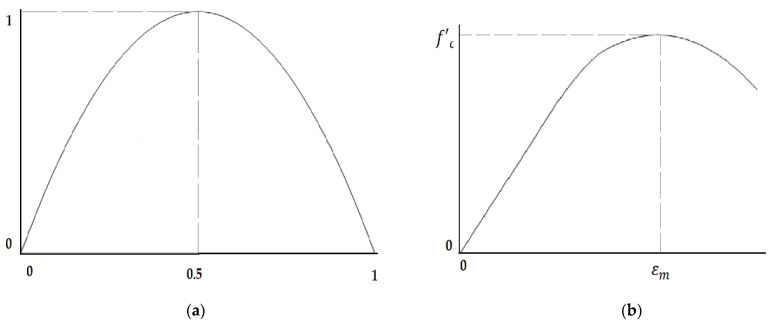
The similarity between (**a**) Logistic Map; and (**b**) the ideological compressive stress–strain curve.

Due to the absolute similarity between the compressive stress–strain curve and the Logistic Map, the Logistic Function and the Map were chosen to predict the compressive strength and simulate the compressive stress–strain curves. This similarity can be seen in the concept of the Logistic Map (Figure 7a) and the conception of the compressive stress–strain curve and the ideological stress–strain curve (Figure 7b).

The Logistic Function and Map ($L_{\mu \;}:\mathbb{R}\to \mathbb{R}$) is explained in Equation (1), where u (Real Parameter) is defined as the Real Parameter with a range between 1 and 4. Moreover, if the μ>4, then the range of the dynamic systems of $L_{\mu }$ (Logistic Function) is not a subset between 0 and 1 [33].

Equation (1) is defined as the Logistic Function. Goodson [33] defined the Logistic Function as the following:

(1) $$L_{\mu }\left(x\right)=\mu x\left(1-x\right),$$

Equation (2) follows Equation (1), where a is the assumption of the maximum domain. Continuing from this, Equation (2) $L_{\mu }$ defines the domain of (0,a). Equation (3) explains the a  as the $\varepsilon_{t}$ imaginary total strain parameter, where $\varepsilon_{m}$ is defined as the strain of the compressive strength. The compressive strength $\left(f{\;}_{c}\right)$ is redefined as $\sigma_{m}$ in Equation (4). Equations (2)–(4) are explained as follows:

(2) $$L_{\mu }\left(x\right)=\mu x\left(a-x\right),$$

(3) $$a=\;\varepsilon_{t}=2\varepsilon_{m},$$

(4) $$f^{\prime }c=\sigma_{m},$$

Finally, Equations (4) and (5) are explained as the Real Parameter u of the Logistic Function in Equation (6). Equations (5)–(7) are defined as follows:

(5) $$L_{\mu }\left(\varepsilon_{m}\right)=\mu \varepsilon_{m}\left(\varepsilon_{t}-\varepsilon_{m}\right),$$

(6) $$\sigma_{m}=\mu \varepsilon_{m}\left(\varepsilon_{t}-\varepsilon_{m}\right),$$

(7) $$\mu =\frac{\sigma_{m}}{\varepsilon_{m}\left(\varepsilon_{t}-\varepsilon_{m}\right)},$$

By including the strain values into Equations (5)–(7) and calculating the required values, the compressive strength of each point was also obtained. The final compressive stress–strain curve was plotted by connecting all of the predicted points. The generated compressive stress–strain curve was similar to the Logistic Map (Figure 7b).

**Figure 8:**
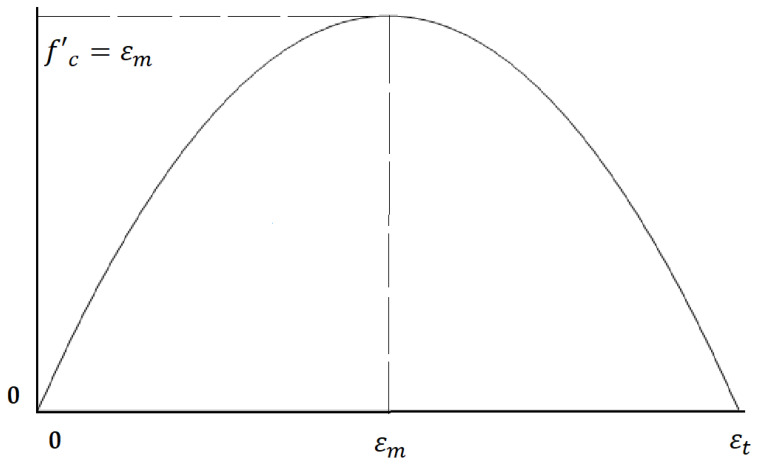
The conception of ideological compressive strength of the stress–strain curve is considered by the Logic Map.

To clarify the issue, Figure 8 illustrates all of the prediction steps. First, the $\varepsilon_{t}$ (Imaginary total strain) is calculated by Equation (3). The $\varepsilon_{t}$ is just obtained to divide the compressive stress–strain curve into smaller pieces to predict more compressive stress points. For example, this study used 20 division points to predict better the compressive stress points. The next step is to find the Real Parameter u via Equation (7); this number is constant for each concrete type. It should be noted that, $\sigma_{m}$ as the compressive strength or compressive stress, and $\varepsilon_{m}$ are calculated through the relationship between the compressive strength and the percentage of BF. Finally, $\sigma_{m}$ as the stress of each point and compressive stress were obtained, and the connection of the compressive stress was made into the compressive stress–strain curve.

The logistic function is able to simulate the BFHPC compressive stress–strain curve through the logistic map. It should be noted that the prediction of the compressive stress–strain curve and the compressive strength of BFHPC was independent of the basalt fibers percentage as a variable and dependent on the relationship between the compressive strength graphs. After simulating the logistic map, the maximum value of the compressive stress–strain curve is equal to the compressive strength. The compressive strength is evaluated with the coefficient of determination (R2), and the mean absolute errors (MAE) and root mean square error (RMSE) as reliability methods.

The Logistic Map is able to predict the elastic phase of the compressive stress–strain curves and the normality of the elasto-plastic phase, while the plastic phase cannot be accurately simulated. Nevertheless, the logistic and performance map is a suitable method for predicting compressive strength, compressive stress–strain, and compressive stress points.

### 2.3. Research Methodology

Classical Programming runs with Python code programming and uses Anaconda programming to insert the packages known as Conda packages.as an open source software. Additionally, the Spyder open source software environment (lessened by Massachusetts Institute of Technology) was used for simpler Python implementation. The mechanical properties of the Basalt-Fibred The High-Performance Concrete were derived from previous experiment results [4,17]. In the current study, BF was the only variable percentage that was able to improve the flexural and compressive strengths.

**Figure 9:**
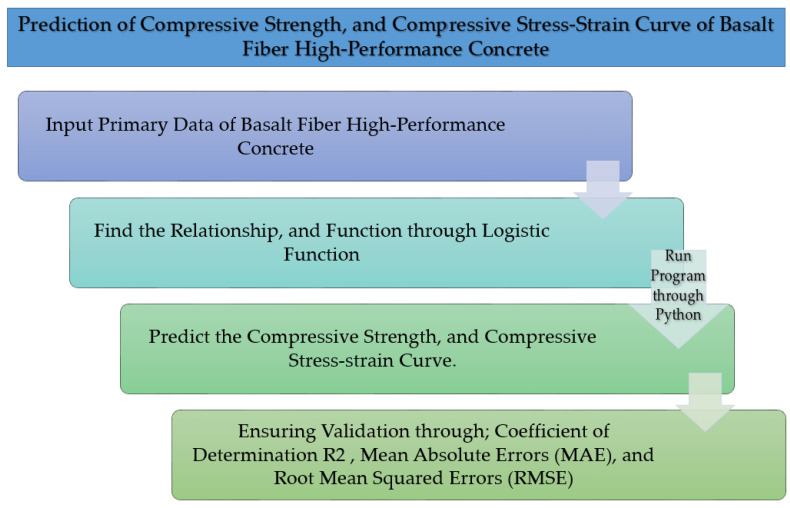
Prediction research methodology in this study.

**Figure 10:**
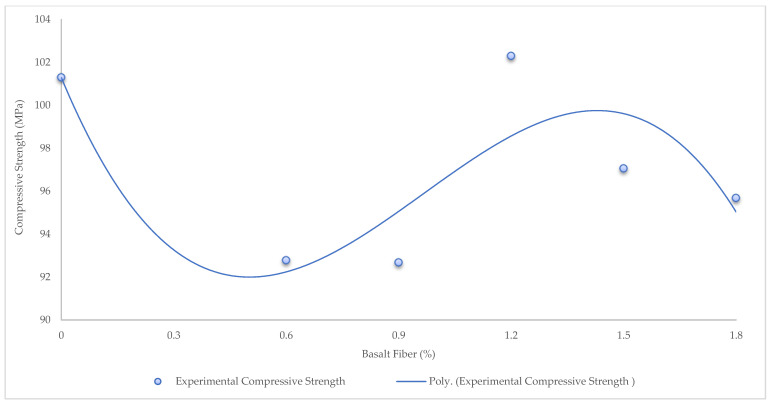
The relationship between BF percentages and compressive strength through Polynomial Function.

The Algorithm was defined to predict the compressive strength and compressive stress–strain (Figure 9). First, the relationship between the BF percentages and compressive strength was obtained through a Polynomial Function (Equation (8)), (Figure 10).

To obtain the relationship between the compressive strength and the BF content percentages, a Polynomial Function was used. The equation of Polynomial Function is mentioned in Equation (8), as follows [34]:

(8) $$y_{\left(x\right)}=\;a_{n}x^{n}+a_{n-1}x^{n-1}+a_{n-2}x^{n-3}+\ldots +a_{1}x^{1}+a_{0},$$

The $a_{n}$ coefficient of a Polynomial Function has a different value under various conditions. $a_{0}$ is the Y-intercept of the Polynomial Function or the Constant value.

The compressive strength prediction was based on real test methods. This means that each piece of concrete is predicted more than 30 times by a random error via Equations (6) and (7). The average of the thirty times prediction results is known as the basic compressive strength. In fact, due to the increase in the statistical population and the increase in the reliability of the prediction, the prediction is performed more than 30 times.

In order to create the prediction algorithm, the compressive stress–strain curve was the basis for the prediction of the compressive strength and the basis for the parameters of the compressive stress–strain prediction algorithm. The Logistic Function parameters were another factor that was effective in predicting the compressive stress–strain curve. Although Logistic Function and Map were only able to predict compressive strength, they were not able to predict other mechanical properties such as flexural and tensile strength.

The compressive stress–strain curves were simulated through the Logistics Map. The absolute similarity between the compressive stress–strain curve and the Logistic Map showed that the compressive stress–strain curve could be found by the Logistic Function. Because of the compressive stress–strain curve simulation, the parameters of the Logistic Function must be found and replaced as the main data in the Python program. The prediction algorithm was attached to a specific sample of concrete. For this study, HPC06 was chosen as the specific specimen. As a result, the compressive stress–strain curves and compressive strength of concrete are possible to predict with this algorithm. Moreover, the prediction of other mechanical properties, such as flexural and compressive strengths, are the limitations of the current algorithm.

The Coefficient of Determination (R2) was applied to find the Algorithm validation and accuracy in this study. Furthermore, the Mean Absolute Errors (MAE) and the Root-Mean-Square Deviation Error (RMSE) are two common types of validation methods to find the reliability of the Algorithm prediction [26]. The MAE equation is equal to the sum of the numerical differences of the values of the community set divided by whole numbers (Equation (9)).

The x, $\hat{x}$, and $\overset{-}{x}$ are the actual, predicted, and mean values of the each community set, respectively, and n is the number of whole sets. The Mean Absolute Errors (MAE)equation is defined as (Equation (9)) [26]:

(9) $$MAE=\frac{1}{n}\sum_{}^{n}\left|x-\hat{x}\right|,$$

The Root-Mean-Square Deviation Error (RMSE) equation is equal to the sum of the numerical differences equal to the power of the two values of the population set divided by the whole set of numbers (Equation (10)). The RMSE equation is explained as (Equation (10)) [26]:

(10) $$RMSE=\sqrt{\frac{1}{n}\sum_{}^{n}{\left(x-\hat{x}\right)}^{2}},$$

Coefficient of Determination (R2) is a measure used in analytics to ensure the correction of the prediction algorithm results, using Equation (11), it is obtained as follows [26]:

(11) $$R^{2}=1-\frac{\sum_{}^{n}{\left(x-\hat{x}\right)}^{2}}{\sum_{}^{n}{\left(x-\overset{-}{x}\right)}^{2}},$$

Generally, Coefficient of Determination (R2), Mean Absolute Errors (MAE), and Root-Mean-Square Deviation Error (RMSE are calculated to obtain the accuracy and reliability of the current algorithm.

### 2.4. OtherPrediction Compressive Stress-Strength Models

To evaluate the validation of the suggested compressive stress–strain curve simulation, some studies were compared with the presented model. The models presented by, Ezeldin and Balaguru [29] and Nataraja et al. [35] were investigated in this study to validate the presented method [27].

Ezeldin and Balaguru [29] studied the prediction of compressive strength and the simulation of stress–strain curves. Equations (12) and (13) are provided to obtain the stress and strain in the stress–strain curve:

(12) $$\sigma {\;}_{c,u}(MPa)=\;\sigma +3.51\left(RI\right)$$

(13) $$\varepsilon {\;}_{c,u}=\varepsilon_{0}\;\;46\times 10^{-6}\left(RI\right)$$

Ezeldin and Balaguru also provide a new method to fit stress–strain: Equations (14) and (15) is shown in the formula:

(14) $$\beta =1.093+713{\left(RI\right)}^{-962}$$

(15) $$RI=V_{f}\frac{I}{d}$$

where $\sigma_{\;}$ is equal to stress,$\;\sigma_{c,u}$, peak stress, $\varepsilon_{c}$ strain, $\varepsilon_{c,u}$ peak strain, $E_{n}$ initial tangent modulus of normal concrete, and β defines the material parameter. In this regard, $V_{f}$, d, and I are the volume fraction, length, and diameter of BF.

The Nataraja et al. models provided another model to find the stress–strain curve. Equations (16)–(19) are explained as:

(16) $$\sigma_{c,u}(MPa)=\sigma +2.16404(RI)$$

(17) $$\varepsilon_{c,u}(MPa)=\varepsilon_{0}+0.0006(RI)$$

(18) $$\beta =0.5811+1.93{\left(RI\right)}^{-0.74}$$

(19) $$E_{inf}=1930{\left(RI\right)}^{-0.74}$$

In this regard, $E_{inf}$ are defined as the slopes at an inflection point.

In general, the two models were used to compare the presented model’s accuracy.

## 3. Results

### 3.1. Experimental Material Results

**Figure 11:**
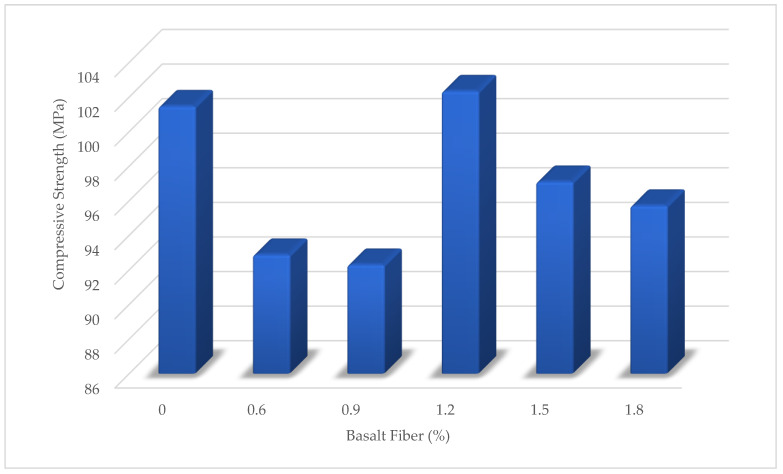
Compressive Strength of BFHPC, With Different BF Percentages.

**Figure 12:**
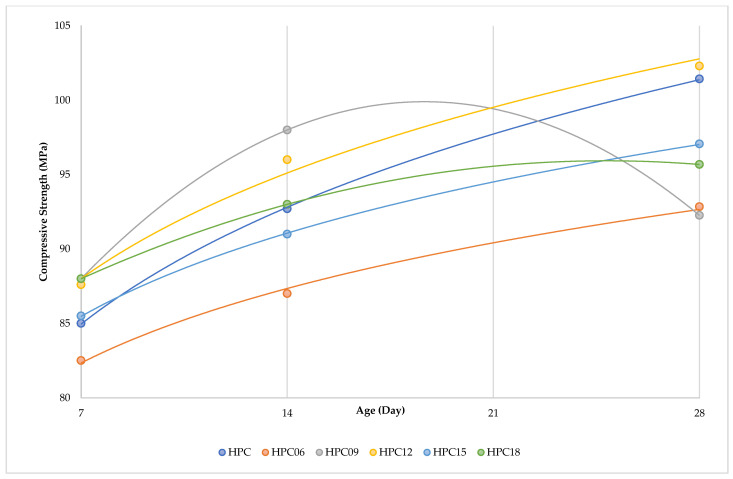
Compressive Strength Workability in Different Curing Periods.

Figure 11 shows the compressive strength results of BFHPC with different BF percentages. The optimal percentage of BF for compressive strength was 1.2. The compressive strength was significantly improved by adding 1.2% of BF. In fact, the improvement in compressive strength of High-Performance Concrete was due to the incorporation of BF and cement matrix at the microscopic scale. Another parameter was the influence of BF volume on compressive strength. Figure 12 shows the effectiveness of concrete at different ages. In fact, all the HPC compressive strength samples increased up to date except for the HPC09 curing period. The HPC09 conditions show that 0.9% BF was not effective at the microscopic scale of the bond between the cement matrix and the fibers.

**Figure 13:**
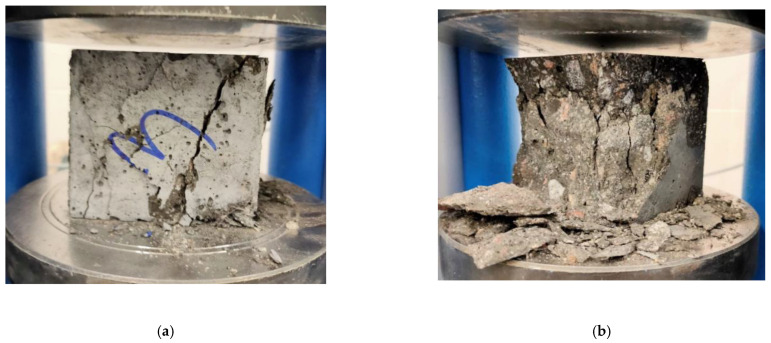
Compression Strength Failure Shapes: (**a**) Basalt Fiber reinforced High-Performance Concrete PC; (**b**) High-Performance Concrete.

For the failure modes of High-Performance Concrete, the compression cube concrete is crushed after loading due to the cyclo–hoop effect, and the shape of the concrete cube block is transferred to a pyramidal mode. (Figure 13a) [3]. High-Performance Concrete with BF had a different failure shape. The BFHPC block is destroyed but not broken completely. The degradation of the BFHPC cube was conducted slowly and crushed very smoothly (Figure 13b).

**Figure 14:**
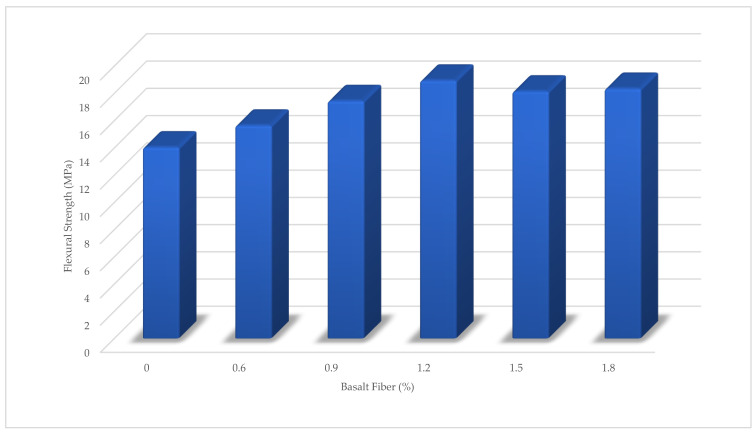
Flexural Strength of BFHPC with different BF Percentages.

**Figure 15:**
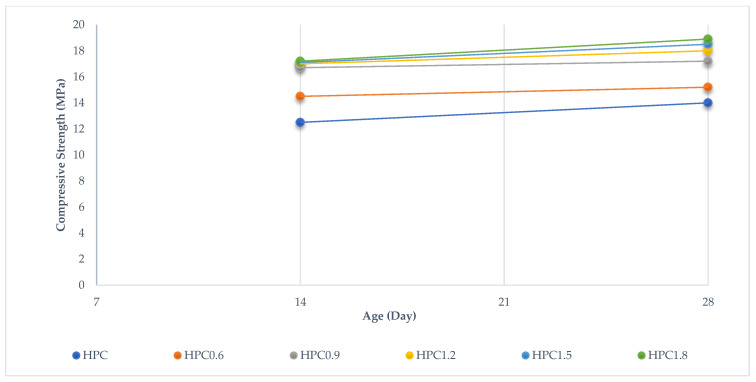
Flexural Strength Workability in Different Curing Periods.

The flexural strength results are shown in Figure 14. The optimal percentage of flexural strength was 1.2%. These conditions show that 1.2% of BF had the greatest effect on the mechanical properties of high-performance concrete. However, the increase in flexural strength was greater than the compressive strength with additional BF, and the main reason for the improvement in flexural strength was the effect of micro-scale bridging phenomena in the cement matrix [4]. The efficiency of flexural strength (Figure 15) shows that the curing period was effective in improving the strength. All samples’ flexural strength improved between 14 and 28 day curing periods.

**Figure 16:**
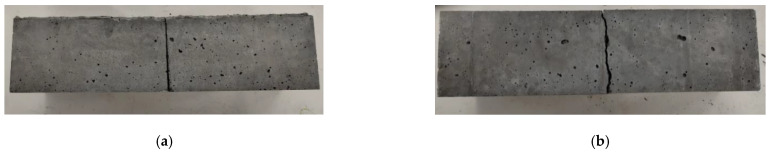
Flexural Strength Failure Shapes: (**a**) Basalt Fiber reinforced High-Performance Concrete; (**b**) High-Performance Concrete.

The failure of the flexural prisms of the specimens for HPC was sudden, while the failure of BFHPC took more time, and the failure model developed after micro-cracks in the prisms (Figure 16).

### 3.2. Compressive Strength Prediction

**Figure 17:**
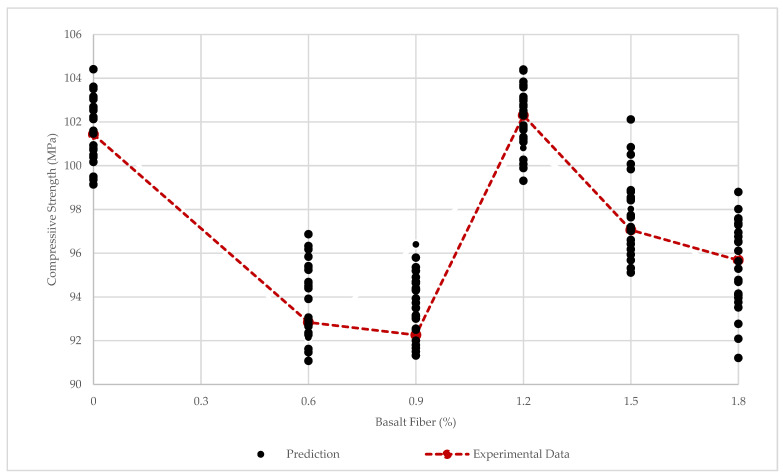
Compressive Strength Prediction, and Experimental Data.

**Figure 18:**
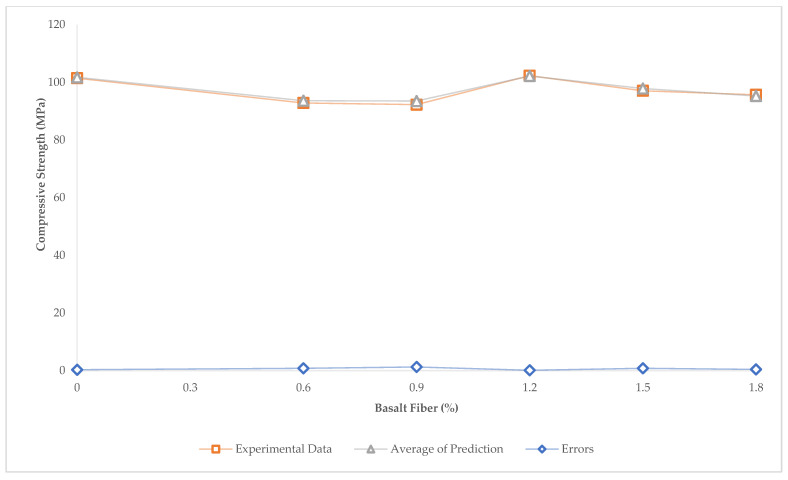
Compressive Strength Average Prediction, Experimental Data, and Errors.

The prediction and experimental compressive strength results are illustrated in Figure 17. According to Figure 17, compressive strengths for more than 30 times prediction were demonstrated. In addition, the prediction errors are shown in Figure 18.

To find the accuracy and reliability, the R2, MAE, and RMSE were calculated between the experimental data and the average of the prediction compressive strengths more than 30 times. The results are shown R2=0.96, MAE=0.59, MSE=0.47. Considering the results, the R2 was close to one, and this means that the current algorithm is reliable and accurate. Another cause of algorithm accuracy is the error value. The maximum error was 1.4 (MPa), and the minimum error was less than 0.103 (MPa) (Figure 18). Thus, the prediction algorithm was able to predict the compressive strength with high accuracy.

### 3.3. The Stress–Strain Curve Prediction

**Figure 19:**
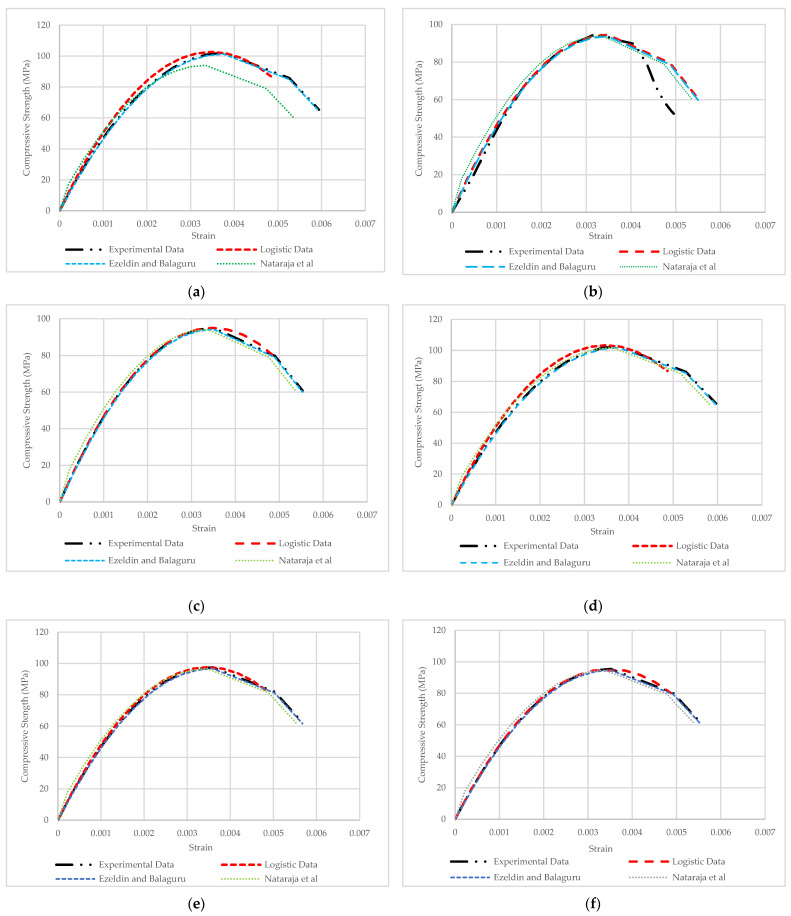
Simulation compressive stress–strain according to Logistic Map, Ezeldin and Balaguru [29], and Nataraja et al. [25]: (**a**) HPC; (**b**) BFHPC0.6; (**c**) BFHPC0.9; (**d**) BFHPC1.2; (**e**) BFHPC1.5; (**f**) BFHPC1.8.

The comparison with Logistic Map simulation is illustrated. The prediction elastic and Elasto-Plastic phases are fitted and varied with high accuracy. The variation between the Logistic Map simulation and the experimental results in the plastic phase is consistent with the nature of the Logistic Function. The plastic phase of the compressive stress–strain curve is an essential property of concrete, reflecting the brittleness and ductility of concrete under compression test. The plastic phase of concrete is not stable, and the durability of concrete is under compression tests. The plastic phase of concrete is not stable and predictable. Nevertheless, the Logistic Map simulates the plastic phase as far as close to the experimental results. Moreover, other compressive stress–strain curve simulation methods are evident that evaluate the Logistic Map curve predictions. The Ezeldin and Balaguru [29] and Nataraja et al. [35] models were employed to evaluate the Logistic Map simulation results. The results presented that not only the behavior of Logistic Map prediction was reasonable, but also, the models were more accurate than Nataraja et al. [35] models (Figure 19).

Thus, it can be inferred from comparing simulations of the stress–strain curve results that the Logistic Map stress–strain results are optimized similar to the two other represented models. The Logistic Map simulation results are following real experiments, which demonstrated the attenuation plastic phase of demonstrating the stress–strain simulation curves through the Logistic Map. Overall, the compressive stress–strain curves were possible to predict via Logistic Map and Function with high accuracy.

## 4. Conclusions

This paper presents a new algorithm for the prediction of compressive strength and compressive stress–strain curve. The current algorithm is used the Logistic Map and Function to find accurate prediction results. In this study, more than 30 times predictions are applied for each mixture design which increased the statistical population to increase the accuracy of prediction. Overall, more than 180 compressive strengths were predicted for all specimens.

The results show that the current algorithm has been successful in predicting compressive strength and compressive stress–strain curve. The results show that the prediction R2, RMSE, and MAE were 0.96, 0.47, and 0.59, respectively. The results prove that the current algorithm was able to present a new, accurate, and easy algorithm to predict compressive strength, and simulate the compressive stress–strain curve. Overall, a general overview is given in the following cases:

Logistic Function was possible to predict with the compressive strength and compressive stress–strain curve for all types of design mixture. However, predicting tensile and flexural strength was not impossible through Logistic Function and Map.

To find the accuracy, the Coefficient of determination (R2), Mean Absolute Errors (MAE), and the Root Mean Squared Errors (RMSE) were applied. R2 was found equal to 0.96, which indicates that the algorithm is reliable. In addition, the RMSE and MAE were 0.47 and 0.57, respectively, which indicated the accuracy of the prediction Algorithm.

The Logistic Map simulated the compressive stress–strain curve with high accuracy so that the presented model was more accurate than Ezeldin and Balaguru [29] and Nataraja et al. [35] models.

The compressive strength and compressive stress–strain curves have been estimated regarding the compressive strength relationship graph. However, the concrete mechanical properties of multi-variable material can predict as well. In fact, the mechanical properties prediction was independent of the number of variables.

The Logistic Map can simulate the Elastic and Elasto-Plastic phases of the compressive stress–strain curve, while the Plastic phase simulation had many errors in this model.

The current Algorithm can be developed considering the Deep Learning programming for the prediction of each type of concrete mechanical behavior.

## Figures and Tables

**Table 1:** Properties of Cement M500.

Oxide (%)								Fineness (m^2^/kg)	Relative Density
SiO_2_	Fe_2_O_3_	MgO	SO_3_	Al_2_O_3_	CaO	K_2_O	LOI		
19.52	4.04	4.36	2.89	4.81	62.18	0.6	1.62	387	3.14

**Table 2:** Properties and Chemical Composition of Micro silica MK-85.

Index	Value (%)
Appearance	Approximate Value
Mass fraction of micro silica in the cross.dryprod.,%	not less than 99.6
Mass fraction of water,%	no more than 0.36
Mass fraction of losses on ignition (pp),%	no more than 0.80
Bulk density, kg/m^3^	152.2
**Chemical Composition**	**Percentage**
Mass fraction of silicon dioxide (SiO_2_)	90–92
Mass fraction of alumina (Al_2_O_3_)	0.68
Mass fraction of iron oxide (Fe_2_O_3_)	0.69
Mass fraction of calcium oxide (CaO)	1.58
Mass fraction of Magnesium oxide (MgO)	1.01
Mass fraction of Sodium oxide (Na_2_O)	0.61
Mass fraction of potassium oxide (K_2_O)	1.23
Mass fraction of Carbon (C)	0.98
Mass fraction of Sulfur (S)	0.26

**Table 3:** Chopped Basalt Fiber Properties.

Length (mm)	Diameter (µm)	Tensile Strength (MPa)	Young’s Modulus (GPa)	Elongation (%)	Specific Gravity
18	17.9	4100–4840	93.1–110	3.1	2.63–2.8

**Table 4:** Concrete mix properties.

Specimens	Cement (Kg/m^3^)	Water (Kg/m^3^)	Granit (Gravel/Couse Aggregate) (Kg/m^3^)	Quartz Sand (Fine Aggregate) (Kg/m^3^)	Micro Silica (Kg/m^3^)	Quartz Flour (Kg/m^3^)	Plasticizing (Kg/m^3^)	Basalt Fiber (%)
**BFHPC**	500	187.5	1005	585	125	100	12.5	_
**BFHPC-6**	500	187.5	1005	585	125	100	12.5	0.6
**BFHPC-9**	500	187.5	1005	585	125	100	12.5	0.9
**BFHPC-1.2**	500	187.5	1005	585	125	100	12.5	1.2
**BFHPC-1.5**	500	187.5	1005	585	125	100	12.5	1.5
**BFHPC-1.8**	500	187.5	1005	585	125	100	12.5	1.8

## Data Availability

Not applicable.
